# Obesity and related comorbidities in a large population-based cohort of subjects with type 1 diabetes in Catalonia

**DOI:** 10.3389/fendo.2022.1015614

**Published:** 2022-12-02

**Authors:** Idoia Genua, Josep Franch-Nadal, Elena Navas, Manel Mata-Cases, Gabriel Giménez-Pérez, Bogdan Vlacho, Didac Mauricio, Albert Goday

**Affiliations:** ^1^ Department of Endocrinology and Nutrition, Hospital de la Santa Creu i Sant Pau, Barcelona, Spain; ^2^ Department of Medicine, Autonomous University of Barcelona, Barcelona, Spain; ^3^ Institut d’Investigació Biomèdica Sant Pau (IIB SANT PAU), Barcelona, Spain; ^4^ Diabetis en Atenció Primaria-Catalunya (DAP-Cat) group, Unitat de Suport a la Recerca Barcelona, Fundació Institut Universitari per a la recerca a l’Atenció Primària de Salut Jordi Gol i Gurina (IDIAPJGol), Barcelona, Spain; ^5^ Primary Health Care Center Raval Sud, Gerència d’Atenció Primaria, Institut Català de la Salut, Barcelona, Spain; ^6^ Centro de Investigación Biomédica en Red (CIBER) of Diabetes and Associated Metabolic Diseases (CIBERDEM), Instituto de Salud Carlos III (ISCIII), Barcelona, Spain; ^7^ Unitat de Suport a la Recerca|, Barcelona, Spain; ^8^ Primary Health Care Center La Mina, Gerència d’Atenció Primària Barcelona Ciutat, Institut Català de la Salut, Sant Adrià de Besòs, Spain; ^9^ Endocrinology Section, Department of Medicine, Hospital General de Granollers, Granollers, Spain; ^10^ School of Medicine and Health Sciences, Universitat Internacional de Catalunya, Sant Cugat del Vallès, Spain; ^11^ Department of Medicine, Universitat de Vic - Universitat Central de Catalunya, Barcelona, Spain; ^12^ Deparment of Endocrinology & Nutrition, Hospital del Mar, IMIM Institut Mar d’Investigacions Mediques. Parc de Salut Mar, Barcelona, Spain; ^13^ CIBERobn, Instituto de Salud Carlos III (ISCIII), Madrid, Spain

**Keywords:** Obesity, type 1 diabetes, cardiovascular risk factors, metabolic syndrome, comorbidities

## Abstract

**Introduction:**

Obesity, an increasing global health problem, can affect people with other disease conditions. The prevalence of obesity in people with type 1 diabetes (T1D) is not well known. The aim of this study was to describe extensively the characteristics and prevalence of different classes of obesity according to BMI (body mass index) categories in a large cohort of patients with T1D.

**Material and methods:**

This was a retrospective, cross-sectional study in Catalonia. We reviewed all patients with T1D diagnosis, ≥ 18 years old and with BMI data from the SIDIAP database. Sociodemographic and clinical data, cardiovascular risk factors, laboratory parameters and concomitant medications were collected.

**Results:**

A total of 6,068 patients with T1D were analyzed. The prevalence of obesity in the total sample was 18% (13.8% with class 1 obesity [BMI 30-34.9 kg/m2]). Patients with obesity had a higher prevalence of other cardiovascular risk factors (i.e. hypertension was 61.4% vs. 37.5%; dyslipidemia 63.6% vs 44%, and chronic kidney disease 38.4% vs. 24.4%; p<0.001 in all cases) and poorer control of them. The higher prevalence was regardless of sex, age and duration of diabetes. The increase in these comorbidities was noticeable from a BMI > 25 kg/m2. Patients with obesity did not have poorer glycemic control.

**Conclusion:**

The presence of obesity in people with T1D is frequent and cardiovascular risk factors are more common and more poorly controlled in T1D patients with obesity.

## Introduction

While it is well-known that obesity is commonly linked to type 2 diabetes (T2D), obesity may also be associated with type 1 diabetes (T1D) in a bidirectional way (1). With the ongoing obesity epidemic and the improvement in T1D treatments, the concern about possible consequences of this relationship has increased in the recent years.

The prevalence of obesity in patients with T1D is not well known. Furthermore, it differs widely among the available studies, varying from 8.9% to 28.4% across large cohort studies made in the USA and west and northern European countries (2–7), and reported to be only 2% in a smaller cohort in an Asian population (8). These variations can probably be explained because the incidence and prevalence of both conditions in the general population vary by country and by regions and, also, the fact that the prevalence of both T1D and obesity has increased in the last decades in the general population (9–11). It is therefore important to have current, region-specific data. Overall, the association between obesity and T1D appears to be frequent and the current prevalence of different classes of obesity in T1D and its characteristics need to be further studied.

The deleterious effect of obesity in the general population is well described (12). Nevertheless, data about the characteristics and consequences in patients with T1D are scarce. The association of obesity in people with T1D may entail additional problems such as poor metabolic control, increased cardiovascular (CV) risk factors and a consequent increase in related complications (13).

In a previous study (14), we described the clinical characteristics of a large cohort of patients with T1D in Catalonia, finding a high prevalence of obesity (16.5%). However, the different degrees of obesity, its characteristics and its implications were not addressed in that initial study. Considering its prevalence and its possible implications, we strongly felt that a more comprehensive analysis was needed; this led us to plan the current sub-study.

The aim of this study was to describe extensively the characteristics and prevalence of obesity (including according to different degrees of obesity) in a large population-based cohort of patients with T1D living in a Mediterranean area, i.e. Catalonia, with a high prevalence of obesity in the general population, and to analyze the impact of the obesity on metabolic control and other CV risk factors.

## Material and methods

We performed a cross-sectional analysis of retrospective routinely collected pseudo-anonymized health data from people who attended primary healthcare centers from the leading public healthcare provider (Institut Català de la Salut, ICS) in Catalonia (Spain). This pseudo-anonymized health data was obtained from the SIDIAP database (*Sistema d’Informació per al desenvolupament de la Investigació en Atenció Primària*, https://www.sidiap.org/), which is a well validated primary healthcare database used in diabetes epidemiological research (14). The SIDIAP database collects healthcare data from more than 74% (5,564,292 persons) of the Catalonian population. The cross-sectional analysis was performed on the cut-off date (December 31st, 2016) for 12 months (from January 1st, 2016).

### Study participants

For our analysis, we included all of the subjects in the database with a diagnosis of T1D, defined as the presence of the diagnostic ICD-10 (International Classification of Diseases 10) code E10 and sub-codes, as described in the previous study (14). Only patients ≥18 years old and with body mass index (BMI) data (directly measured and recorded by a healthcare professional) were considered for the analysis. Those with an E10 diagnosis treated with any glucose-lowering agents other than insulin and those who had not been treated with short-acting insulins for more than two years after the recorded date of diagnosis were excluded, as described previously (14).

No formal sample size calculation was done, as the current study is a sub-analysis of the initial study on the characteristics of type 1 diabetes in Catalonia (14).

### Study variables

On the cut-off date, we collected different sociodemographic data (age, sex), clinical data (BMI, blood pressure, age at T1D diagnosis, duration of T1D), CV risk factors (hypertension, dyslipidemia, smoking, and obesity), laboratory parameters (HbA1c, blood lipids, estimated glomerular filtration rate (GFR; using the CKD-EPI formula) and insulin therapy and other concomitant medications related to CV risk factors. Dyslipidemia and hypertension were defined by the ICD-10 diagnostic code and/or a record of lipid-lowering or antihypertensive drugs, respectively. Chronic kidney disease (CKD) was defined by the ICD-10 diagnostic code and/or GFR <60 or urine albumin to creatinine ratio (ACR) >30 mg/g. Diagnostic codes and proxies used to define the study variables are presented in Supplementary Appendix 1 (code list). Objectives for CV prevention were established according to the European Society of Cardiology guidelines (15) (HbA1c <7% and blood pressure <140/90 mmHg and low-density lipoprotein cholesterol (LDLc) <100 mg/dl for primary prevention and HbA1c <7% and blood pressure <140/90mmHg and LDLc <70mg/dl for secondary prevention).

A complete-case analysis was performed.

### Statistical analysis

Descriptive statistics were calculated to summarize the sociodemographic characteristics, as well as the study variables (clinics and treatments). Continuous variables were expressed as means and standard deviations or medians and interquartile range, and categorical variables were expressed as frequencies and percentages. Descriptive analyses were conducted for the overall population, and stratified by sex and BMI groups (<30, 30 – 34.9, 35 – 39.9 and ≥40 kg/m^2^) for all the study variables. In addition, for the clinical variables, analyses were stratified by sex, age groups (<40 and ≥40 years) and diabetes duration (<10, 10–19 and >19 years). To evaluate the differences between the characteristics (variables) according to the generated groups, the Chi-square test and the F-Fisher test were used when appropriate. To analyze continuous variables, the tests used were the T-Student test or analysis of variance (Anova). Additionally, the Bonferroni test was performed for multiple comparisons. To obtain the optimal cut-off points of the BMI in the ROC analysis, the “Concordance Probability Method (CZ)” was used. Statistical analysis was performed using R software for Windows version 3.6.1, Vienna, Austria.

## Results

A description of the whole cohort of patients with T1D in Catalonia has been published previously by this group elsewhere (14). From the overall number of subjects with T1D (15,008), we included those older than 18 years old (13,547) who had BMI data available (6,610). A total of 6,068 patients with T1D were analyzed in the current study. The sociodemographic and clinical characteristics of the studied population according to sex are shown in **Table 1**. The mean age of the entire cohort was 48.7 ± 15.9 years old with mean glycosylated hemoglobin (HbA1c) value of 8.0 ± 1.4%. The mean age at T1D diagnosis was 35.0 years, and 54.6% were men. Only 8.3% of the patients achieved all 3 risk factor target level for primary prevention for cardiovascular diseases (HbA1c <7%, BP<140/90 and LDLc <100 mg/dl).

**Table 1:** Characteristics of the whole study population and also according to sex.

	Total population	Women	Men	p-value
N	6068 (100.0%)	2756 (45.4%)	3312 (54.6%)	
Current age (years)	48.7 (15.9)	49.3 (16.8)	48.3 (15.1)	0.272
Age at diagnosis (years)	35.0 (17.6)	35.3 (18.9)	34.8 (16.4)	>0.999
**Diabetes duration (years)**				>0.999
<10 years	2084 (34.3%)	925 (33.6%)	1159 (35.0%)	
10 – 19 years	2703 (44.5%)	1227 (44.5%)	1476 (44.6%)	
>19 years	1281 (21.1%)	604 (21.9%)	677 (20.4%)	
HbA1c % (n = 4249)	8.0 (1.4)	8.0 (1.4)	8.0 (1.4)	>0.999
**BMI (kg/m^2^)** (n= 6068)	26.2 (4.5)	26.1 (5.0)	26.2 (4.1)	**0.003**
Non obesity (BMI < 30 kg/m^2^)	4976 (82.0%)	2202 (79.9%)	2774 (83.8%)	
Obesity (BMI ≥ 30 kg/m^2^)	1092 (18.0%)	554 (20.1%)	538 (16.2%)	
**Smoking status**:				**<0.001**
Non-smoker	2885 (47.5%)	1598 (58.0%)	1287 (38.9%)	
Ex-smoker	1638 (27.0%)	625 (22.7%)	1013 (30.6%)	
Smoker	1545 (25.5%)	533 (19.3%)	1012 (30.6%)	
**BP**				
Systolic BP (mmHg) (n= 5711)	127 (14.0)	125 (14.7)	129 (13.0)	**<0.001**
Diastolic BP (mmHg) (n= 5711)	73.6 (9.1)	72.4 (8.8)	74.6 (9.2)	**<0.001**
Total cholesterol (mg/dl) (n=4331)	182 (37.9)	187 (36.4)	178 (38.7)	**<0.001**
HDLc (mg/dl) (n= 4145)	59.1 (17.3)	65.4 (17.2)	53.7 (15.5)	**<0.001**
LDLc (mg/dl) (n= 3933)	103 (30.2)	103 (29.0)	102 (31.2)	>0.999
Triglycerides (mg/dl) (n= 4005)	111 (91.3)	100 (64.3)	120 (108.0)	**<0.001**
Microalbuminuria (n= 3252)	68.2 (275)	61.5 (278)	74.0 (272)	>0.999
Estimated GFR (CKDEPI) (n= 3632)	79.2 (22.4)	78.4 (22.3)	79.9 (22.4)	0.929
**Other variables**				
Chronic kidney disease (n= 2962)	805 (27.2%)	366 (26.9%)	439 (27.4%)	>0.999
Dyslipidemia (%), (n= 6068)	2881 (47.5%)	1225 (44.4%)	1656 (50.0%)	**<0.001**
Hypertension (%), (n= 6068)	2534 (41.8%)	1082 (39.3%)	1452 (43.8%)	**0.009**
**Cardiovascular prevention**				
Primary (%), (n = 4434)	368 (8.3%)	170 (8.5%)	198 (8.2%)	>0.999
Secondary (%), (n = 4434)	114 (2.5%)	38 (1.8%)	76 (3.1%)	0.256
**More treatments**				
BP lowering (%)	2366 (39.0%)	1005 (36.5%)	1361 (41.1%)	**0.006**
Antithrombotic (%)	1675 (27.6%)	673 (24.4%)	1002 (30.3%)	**<0.001**
Lipid-lowering (%)	2500 (41.2%)	1055 (38.3%)	1445 (43.6%)	**<0.001**
**Insulin therapy**				
Short-acting insulin (%)	5603 (92.3%)	2576 (93.5%)	3027 (91.4%)	0.068
Long-acting insulin (%)	5093 (83.9%)	2275 (82.5%)	2818 (85.1%)	0.198
Mixed insulin (%)	873 (14.4%)	378 (13.7%)	495 (14.9%)	>0.999
Intermediate-acting insulin (%)	316 (5.2%)	135 (4.9%)	181 (5.4%)	>0.999

P-value<0.05 is considered to be statistically significant. Data are mean (SD) or n(%). Where indicated, the n value denotes the number of study patients with available data during the study period. BMI, body mass index; BP, blood pressure; HDLc, HDL-cholesterol; LDLc, LDL-cholesterol; GFR, glomerular filtration rate. Primary prevention includes the composite target achievement of HbA1c<7%, blood pressure <140/90 and LDL-C<100. Secondary prevention includes the composite target achievement of HbA1c<7%, blood pressure <140/90 and LDL-C<70. Bold values correspond to statistically significant p-values.

The mean BMI was 26.2 ± 4.5 kg/m^2^, with a prevalence of obesity (BMI ≥30 kg/m^2^) of 18%. There were no statistically significant differences between sexes (20.1% in women and 16.2% in men). Regarding different degrees of obesity, 13.7% of patients with T1D had class 1 obesity (BMI between 30-34.9 kg/m^2^), 3.3% had class 2 obesity (BMI between 35-39.9 kg/m^2^) and only 1% had class 3 obesity (BMI ≥40 kg/m^2^) (**Table 2**).

**Table 2:** Characteristics of T1D according to the different categories of body mass index.

	No Obesity< 30kg/m^2^	Obesity Class 130-34.9 kg/m^2^	Obesity Class 235-39.9 kg/m^2^	Obesity Class 3≥ 40 kg/m^2^	p-value
N (%)	4976 (82.0%)	830 (13.8%)	199 (3.2%)	63 (1.0%)	
Current age (years)	47.7 (15.7)	53.0 (15.7)	55.9 (15.9)	53.7 (16.6)	**<0.001**
Age at diagnosis (years)	34.1 (17.2)	38.4 (18.4)	42.5 (19.6)	41.9 (19.1)	**<0.001**
**Diabetes duration (years)**					>0.999
<10 years	1722 (34.6%)	258 (31.1%)	80 (40.2%)	24 (38.1%)	
10 – 19 years	2221 (44.6%)	380 (45.8%)	73 (36.7%)	29 (46.0%)	
>19 years	1033 (20.8%)	192 (23.1%)	46 (23.1%)	10 (15.9%)	
HbA1c % (n = 4249)	8.0 (1.5)	7.9 (1.3)	8.1 (1.3)	8.1 (1.6)	>0.999
BMI (kg/m^2^) (n= 6068)	24.6 (2.9)	31.9 (1.3)	36.9 (1.4)	43.3 (3.2)	**<0.001**
**Smoking status**					**<0.001**
Non smoker	2321 (46.6%)	422 (50.8%)	105 (52.8%)	37 (58.7%)	
Former Smoker	1209 (24.3%)	252 (30.4%)	68 (34.2%)	16 (25.4%)	
Smoker	1446 (29.1%)	156 (18.8%)	26 (13.1%)	10 (15.9%)	
**BP**					
Systolic BP (mmHg) (n= 5711)	126 (13.9)	131 (13.3)	131 (12.4)	132 (16.6)	**<0.001**
Diastolic BP (mmHg) (n= 5711)	73.2 (9.0)	75.3 (9.2)	75.5 (9.2)	74.2 (12.0)	**<0.001**
Total cholesterol (mg/dl) (n=4331)	183 (37.6)	180 (38.0)	179 (41.0)	194 (48.0)	0.709
HDLc (mg/dl) (n= 4145)	60.5 (17.5)	53.6 (15.3)	51.2 (15.1)	51.8 (13.5)	**<0.001**
LDLc (mg/dl) (n= 3933)	103 (30.1)	102 (31.4)	98.8 (30.6)	103 (25.2)	>0.999
Triglycerides (mg/dl) (n= 4005)	106 (84.3)	129 (119)	141 (82.9)	164 (122)	**<0.001**
Microalbuminuria (n= 3252)	64.7 (273)	64.5 (251)	108 (301)	224 (452)	**0.024**
Estimated GFR (CKDEPI) (n= 3632)	80.4 (21.7)	76.4 (23.3)	69.1 (24.3)	62.9 (28.8)	**<0.001**
**Other variables**					
Chronic kidney disease (n= 2962)	577 (24.4%)	155 (34.9%)	51 (46.8%)	22 (53.7%)	**<0.001**
Dyslipidemia (%), (n= 6068)	2187 (44.0%)	519 (62.5%)	135 (67.8%)	40 (63.5%)	**<0.001**
Hypertension (%), (n= 6068)	1864 (37.5%)	485 (58.4%)	139 (69.8%)	46 (73.0%)	**<0.001**
**Cardiovascular Prevention**					
Primary (%), (n = 4434)	303 (8.5%)	54 (8.3%)	9 (5.9%)	2 (3.9%)	>0.999
Secondary (%), (n = 4434)	86 (2.4%)	24 (3.7%)	4 (2.6%)	0 (0.0%)	>0.999
**More treatments**					
BP lowering (%)	1735 (34.9%)	455 (54.8%)	132 (66.3%)	44 (69.8%)	**<0.001**
Antithrombotic (%)	1259 (25.3%)	307 (37.0%)	80 (40.2%)	29 (46.0%)	**<0.001**
Lipid-lowering (%)	1868 (37.5%)	472 (56.9%)	124 (62.3%)	36 (57.1%)	**<0.001**
**Insulin therapy**					
Short-acting insulin (%)	4626 (93.0%)	747 (90.0%)	176 (88.4%)	54 (85.7%)	**0.016**
Long-acting insulin (%)	4220 (84.8%)	672 (81.0%)	155 (77.9%)	46 (73.0%)	**0.008**
Mixed insulin (%)	673 (13.5%)	144 (17.3%)	37 (18.6%)	19 (30.2%)	**0.001**
Intermediate-acting insulin (%)	258 (5.1%)	42 (5.0%)	12 (6.0%)	4 (6.3%)	>0.999

P-value<0.05 is considered to be statistically significant. Data are mean (SD) or n(%). Where indicated, the n value denotes the number of study patients with available data during the study period. BMI, body mass index; BP, blood pressure; GFR, glomerular filtration rate; HDLc, HDL-cholesterol; LDLc, LDL-cholesterol. Primary prevention includes the composite target achievement of HbA1c<7%, blood pressure <140/90 and LDL-C<100. Secondary prevention includes the composite target achievement of HbA1c<7%, blood pressure <140/90 and LDL-C<70. Bold values correspond to statistically significant p-values.

Compared to patients with T1D without obesity, those with obesity were older at the time of the study (53.6 ± 15.8 vs 47.7 ± 15.7 years old; p<0.001), and also at the time of T1D diagnosis (39.4 ± 18.7 vs 34.1 ± 17.2 years old; p<0.001), but there was no difference in the duration of T1D from diagnosis (14.8 vs 14.2 years). The prevalence of obesity gradually increased with age (6.2% in ≤20 years old, 9.7% in 21-30 years old, 14.6% in 31-40 years old, 16.1% in 41-50 years old, 20.9% in 51-60 years old, 21.8% in 61-70 years old and 29.6% in >70 years old; p<0.001). Patients with obesity were more likely to be non-smokers.

Concerning glycemic control, patients with obesity did not have a poorer control compared to those without obesity (mean HbA1c 8.0 ± 1.3 vs 8.0 ± 1.5, respectively; p=0.233). Moreover, having obesity did not make it less likely to have glycemic control within the optimal range (defined as HbA1c <7%), with 21.9% of patients with obesity and 21.5% without obesity having HbA1c < 7%.

**Table 2** shows the clinical characteristics of patients with T1D according to different degrees of obesity. Compared to non-obese patients, a higher proportion of those with obesity had CV risk factors (hypertension was 61.4% vs. 37.5%; dyslipidemia 63.6% vs 44%, and chronic kidney disease 38.4% vs. 24.4%; p<0.001 in all cases). Furthermore, obesity was associated with poorer control of some CV risk factors; fewer patients with obesity than those without had fair blood pressure control (defined as <140/90 mmHg) (75% vs. 84% (p <0.001), triglyceride levels were higher in the obese group (134 ± 113 vs 106 ± 84.3 mg/dl; p<0.001), and high density lipoprotein cholesterol (HDLc) levels were lower in the obese group (53.1 ± 15.2 vs 60.5 ± 17.5 mg/dl; p<0.001). No differences were found in total cholesterol or LDLc levels. We have also compared CV risk factors of patients with T1D according to the different obesity categories, and the same differences were observed, as shown in **Table 2**.

No differences were found in the percentage of patients that met the established targets for primary (HbA1c <7% and blood pressure <140/90 and LDLc <100 mg/dl) and secondary (HbA1c <7% and blood pressure <140/90 and LDLc <70 mg/dl) CV prevention when comparing distinct BMI categories.

The prevalence of obesity and clinical characteristics according to gender, current age and diabetes duration are presented in **Tables 3A**–**C**. The higher prevalence of hypertension and dyslipidemia in the obesity group was maintained irrespective of sex, age and duration of diabetes.

**Table 3A:** Prevalence of obesity and clinical characteristics according to sex.

Sex						
	Women (n = 2756)			Men (n = 3312)		
	<30kg/m^2^	≥30kg/m^2^	p-value	<30kg/m^2^	≥30kg/m^2^	p-value
n (%)	2,202 (79.8%)	554 (20.2%)		2774 (83.7%)	538 (16.2%)	
Systolic BP, mmHg	123 (14.5)	130 (14.1)	**<0.001**	129 (13.0)	132 (12.4)	**<0.001**
Diastolic BP, mmHg	72.0 (8.7)	73.7 (9.4)	**<0.001**	74.1 (9.2)	76.8 (9.1)	**<0.001**
Total cholesterol (mg/dl)	188 (36.2)	185 (37.1)	>0.999	178 (38.2)	176 (41.2)	>0.999
HDLc (mg/dl)	67.4 (17.2)	58.0 (15.0)	**<0.001**	54.9 (15.6)	47.8 (13.5)	**<0.001**
LDLc (mg/dl)	103 (28.8)	103 (29.8)	>0.999	103 (31.1)	99.9 (32.0)	>0.999
Triglycerides (mg/dl)	94.7 (60.5)	120 (72.9)	**<0.001**	114 (98.2)	148 (143)	**<0.001**
HbA1c %	8.0 (1.5)	8.0 (1.3)	>0.999	8.0 (1.5)	7.9 (1.3)	>0.999
Dyslipidemia	903 (41.0%)	322 (58.1%)	**<0.001**	1284 (46.3%)	372 (69.1%)	**<0.001**
Hypertension	756 (34.3%)	326 (58.8%)	**<0.001**	1108 (39.9%)	344 (63.9%)	**<0.001**
Chronic kidney disease	239 (22.7%)	127 (41.5%)	**<0.001**	338 (25.7%)	101 (35.1%)	**0.018**

BP, blood pressure, HbA1c, glycosylate haemoglobin; HDLc, HDL-cholesterol; LDLc, LDL-cholesterol. P-value<0.05 is considered to be statistically significant. Bold values correspond to statistically significant p-values.

**Table 3B:** Prevalence of obesity and clinical characteristics according to current age.

Current age (years)						
	<40 years (n = 1843)			≥40 years (n = 4225)		
	<30kg/m^2^	≥30kg/m^2^	p-value	<30kg/m^2^	≥30kg/m^2^	p-value
N (%)	1618 (87.8%)	225 (12.2%)		3358 (79.4%)	867 (20.6%)	
Systolic BP, mmHg	121 (12.6)	125 (12.0)	**<0.001**	129 (13.8)	133 (13.2)	**<0.001**
Diastolic BP, mmHg	72.9 (8.6)	77.9 (7.3)	**<0.001**	73.4 (9.2)	74.6 (9.7)	**0.007**
Total cholesterol (mg/dl)	184 (39.1)	183 (36.8)	>0.999	182 (36.9)	180 (40.0)	>0.999
HDLc (mg/dl)	59.4 (15.3)	51.3 (13.6)	**<0.001**	61.0 (18.3)	53.5 (15.5)	**<0.001**
LDLc (mg/dl)	105 (30.5)	108 (30.1)	>0.999	102 (29.9)	99.9 (30.9)	>0.999
Triglycerides (mg/dl)	104 (88.2)	133 (187)	0.618	106 (82.5)	132 (86.6)	**<0.001**
HbA1c %	8.2 (1.7)	7.9 (1.3)	**0.028**	8.0 (1.3)	8.0 (1.3)	>0.999
Dyslipidemia	200 (12.4%)	53 (23.6%)	**<0.001**	1987 (59.2%)	641 (73.9%)	**<0.001**
Hypertension	154 (9.5%)	45 (20.0%)	**<0.001**	1710 (50.9%)	625 (72.1%)	**<0.001**
Chronic kidney disease	87 (12.7%)	18 (17.1%)	>0.999	490 (29.1%)	210 (42.9%)	**<0.001**

BP, blood pressure; HbA1c, glycosylate haemoglobin; HDLc, HDL-cholesterol; LDLc, LDL-cholesterol. P-value<0.05 is considered to be statistically significant. Bold values correspond to statistically significant p-values.

**Table 3C:** Prevalence of obesity and clinical characteristics according to diabetes duration.

Diabetes duration (years)	<10 years (n = 2084)			10 – 19 years (n = 2703)			>19 years (n = 1281)		
	<30kg/m^2^	≥30kg/m^2^	p-value	<30kg/m^2^	≥30kg/m^2^	p-value	<30kg/m^2^	≥30kg/m^2^	p-value
N(%)	1722 (82.7%)	362 (17.3%)		2221 (82.1%)	482 (17.9%)		1033 (80.6%)	248 (19.4%)	
Systolic BP, mmHg	125 (14.6)	131 (14.4)	**<0.001**	127 (13.7)	132 (12.6)	**<0.001**	128 (13.1)	131 (13.2)	**0.036**
Diastolic BP, mmHg	72.8 (9.0)	75.7 (9.3)	**<0.001**	73.6 (8.9)	75.5 (9.5)	**<0.001**	73.0 (9.3)	74.2 (9.2)	>0.999
Total cholesterol (mg/dl)	183 (39.2)	181 (41.0)	>0.999	183 (36.6)	182 (38.1)	>0.999	181 (36.9)	179 (39.4)	>0.999
HDLc (mg/dl)	58.7 (17.6)	50.2 (14.3)	**<0.001**	61.3 (17.2)	53.7 (15.1)	**<0.001**	62.0 (17.7)	56.3 (15.9)	**<0.001**
LDLc (mg/dl)	103 (31.4)	103 (30.7)	>0.999	103 (29.7)	103 (31.3)	>0.999	100 (28.4)	95.3 (29.8)	0.725
Triglycerides (mg/dl)	114 (108)	154 (157)	**<0.001**	102 (69.7)	123 (76.1)	**<0.001**	99.0 (60.5)	123 (85.3)	**0.008**
HbA1c %	8.0 (1.7)	7.8 (1.5)	0.405	8.1 (1.3)	8.1 (1.3)	>0.999	8.0 (1.3)	7.9 (1.2)	>0.999
Dyslipidemia	640 (37.2%)	211 (58.3%)	**<0.001**	980 (44.1%)	301 (62.4%)	**<0.001**	567 (54.9%)	182 (73.4%)	**<0.001**
Hypertension	528 (30.7%)	216 (59.7%)	**<0.001**	808 (36.4%)	277 (57.5%)	**<0.001**	528 (51.1%)	177 (71.4%)	**<0.001**
Chronic kidney disease	201 (23.9%)	102 (48.1%)	**<0.001**	234 (23.6%)	75 (29.4%)	0.675	142 (26.5%)	51 (40.2%)	**0.036**

P-value<0.05 is considered to be statistically significant. BP, blood pressure; HDLc, HDL-cholesterol; LDLc, LDL-cholesterol. Bold values correspond to statistically significant p-values.

**Table 4** represents the relation between BMI, as a continuous variable, and the degree of control of CV risk factors showing a progressive worsening in their control from a BMI of >25 kg/m^2^. The BMI cut-off values at which the progressive worsening occurred were 25.6, 25.6, 25.4, 26.6, 25.2 and 26.5 kg/m^2^ for systolic and diastolic blood pressure, total cholesterol, HDLc, LDLc and triglycerides, respectively.

**Table 4:** Cut-off points of the body mass index that show optimal or suboptimal control according to clinical variables.

Variables	Suboptimal control	Patients with suboptimal control N (%)	Optimal control	Patients with optimal control N (%)	BMI cut-off points	AUC (95%CI)	Sensitivity or True Positive Rate	Specificity or True Negative Rate
Systolic BP, mmHg	≥130 mmHg	2709 (47.4%)	<130 mmHg	3002 (52.5%)	25.6 kg/m^2^	0.62 (0.60; 0.63)	60.1%	57.4%
Diastolic BP, mmHg	≥80 mmHg	1570 (27.4%)	<80 mmHg	4141 (72.5%)	25.6 kg/m^2^	0.58 (0.56; 0.59)	60.3%	52.6%
Total cholesterol (mg/dl)	≥240 mg/dl	307 (7.1%)	<240 mg/dl	4024 (92.9%)	25.4 kg/m^2^	0.50 (0.46; 0.53)	56.4%	46.3%
HDLc (mg/dl)								
Men	<40 mg/dl	351 (15.6%)	≥40 mg/dl	1891 (84.3%)	26.6 kg/m^2^	0.61 (0.57; 0.64)	59.8%	57.9%
Women	<50 mg/dl	329 (17.2%)	≥50 mg/dl	1574 (82.7%)	26.1 kg/m^2^	0.67 (0.63; 0.70)	69.9%	59.4%
LDLc (mg/dl)	≥100 mg/dl	1987 (50.5%)	<100 mg/dl	1946 (49.4%)	25.2 kg/m^2^	0.51 (0.49; 0.53)	57.9%	46.8%
Triglycerides (mg/dl)	≥150 mg/dl	662 (16.5%)	<150 mg/dl	3343 (84.4%)	26.5 kg/m^2^	0.60 (0.58; 0.62)	59.8%	57.7%
Glomerular Filtration (mg/dL)	<60 mg/dL	471 (12.9%)	≥60 mg/dL	3161 (87.0%)	27.6 kg/m^2^	0.60 (0.57; 0.63)	51.6%	66.4%
Microalbuminuria	>30 mg	542 (16.6%)	≤30 mg	2710 (83.3%)	27.2 kg/m^2^	0.53 (0.51; 0.56)	45.8%	62.1%

AUC, area under the curve; BP, blood pressure; CI, confidence interval; HDLc, high-density lipoprotein cholesterol; LDLc, low-density lipoprotein cholesterol; BMI, body mass index.

## Discussion

The present study confirms a high prevalence of obesity in people with T1D, with a concomitant higher prevalence and poorer control of other CV risk factors. The prevalence of T1D in Spain is medium-high, being around 15 cases per 1,000 in people under 19 years old (16) and 0.3% in the working population (17). The estimated prevalence of obesity in Spain is also medium-high at around 23% (18). With the prevalence of both conditions being medium-high in our area, it is relevant to explore the association between both diseases. To our knowledge, this is the largest cohort that describes extensively the prevalence and clinical characteristics of obesity, including different degrees of obesity, in adults with T1D.

The prevalence of obesity observed in patients with T1D (18%, mainly class 1 obesity) is similar or a bit lower than the prevalence of obesity in the general population in Spain (23%) (18). Both epidemiological data are based on BMI according to the direct measurement of weight and height, and therefore are methodologically homogeneous. These data are more accurate than data on self-declaration of weight and height, as obtained in national health surveys. The prevalence found in our cohort is in line with another recently published European study in Belgium in a cohort of 32,809 patients (between 2017 and 2018) with T1D that reported a prevalence of obesity of around 17%, similar to that in the Belgium general population (4). Another recent large European cohort study found a prevalence of obesity of 15.3% in T1D (3). As mentioned in a recent review (1), the prevalence found in the current study is close to the prevalence of obesity seen in the general population. On the other hand, other recent studies in large cohorts from non-European countries with a higher prevalence of obesity in the general population, observed a markedly lower prevalence of obesity in T1D (2, 19). Despite these differences, it is definitely clear that obesity in people with T1D is a prevalent comorbidity that we cannot disregard.

As expected, considering the general population, patients with obesity were older (18). Unlike in the general population, few studies have evaluated the association of obesity with other CV risk factors in people with T1D. A study that assessed this in 11,348 children in the USA, with a 14% prevalence of obesity, also found a higher prevalence of hypertension and dyslipidemia in the obese group, with no differences in HbA1c values (20). In contrast, a Swedish study in 26,125 people with T1D, found higher HbA1c levels in patients with obesity at baseline, as well as higher mean systolic blood pressure (21). Considering that the main cause of mortality in T1D is CV disease, having an increase and poorer control of CV risk factors in those with obesity is a relevant finding. Further, a study found higher major complication outcomes (an aggregate of mortality, coronary artery disease, and renal failure) in T1D patients with metabolic syndrome (22). Interestingly, patients with obesity were more frequently non-smokers, a fact that is favorable and does not further increase the adverse CV risk profile of these subjects.

Whether people with T1D and obesity have poorer glycemic control remains unclear. We did not find differences in chronic glycemic control (HbA1c). The T1D exchange clinic registry (23) found that adolescents who had better metabolic control were at higher risk of obesity. However, a study of the SWEET registry in children and adolescents found that underweight and obese children had significantly higher HbA1c (24). Although these studies were conducted in pediatric populations that might not be comparable to adults, results from the few studies in adult populations are also inconsistent (21, 25). The fact that a more intensive insulin therapy is related to weight gain could go some way to explaining why obesity does not clearly worsen metabolic control (26).

The established cut-off points by WHO for overweight and obesity in the general population are based on the relationship between BMI and morbidity and mortality. However, it is well known that these cut-offs are inaccurate for some groups (27). Nevertheless, we found a good correlation between the classic BMI cut-off points and the risk of comorbidities in this specific subgroup of patients. We acknowledge that obtained AUC, sensitivity and specificity are not very discriminant. However, we did not aim at building a screening tool, but at exploring the threshold upon which the overweight-related risk factor profile was unfavorable. The major strengths of the study are the extensive description of patients and the large cohort used for the analysis. Data are representative and robust, encompassing a large proportion of the Catalonian population, with the SIDIAP database having been used in several other diabetes studies (14, 28, 29). The restrictive criteria used to define the diagnosis of T1D ensured the correct diagnosis of T1D.

The study has some limitations. First, due to the cross-sectional retrospective nature of the study, inherent limitations of this type of study are presented. We cannot establish tendencies or causality. In addition, obesity was frequently not well documented compared to other diseases, and BMI was probably more often recorded in more complicated and older patients. The study relied on the recorded codes without external validation and because of the restrictive definition criteria we may have excluded a few subjects with T1D who were also treated with glucose-lowering agents other than insulin.

In conclusion, the prevalence of obesity in T1D is relevant and is associated with a higher risk of having other CV risk factors with a suboptimal control of them. It seems that glycemic control is not related to the degree of obesity.

## Data availability statement

Restrictions apply to the availability of some or all data generated or analyzed during this study because they were used under license. Requests to access these datasets should be directed to DM, didacmauricio@gmail.com.

## Ethics statement

The studies involving human participants were reviewed and approved by Primary Health Care University Research Institute Jordi Gol (number P17/115). Written informed consent for participation was not required for this study in accordance with the national legislation and the institutional requirements.

## Author contributions

Conceptualization: AG, JF-N and DM; formal analysis: EN; resources and data curation, EN; writing—original draft preparation IG; writing—review and editing: BV, IG, AG, EN, JF-N, GG-P, MM-C and DM; supervision: DM, AG, and JF-N; project administration: JF-N. All authors contributed to the article and approved the submitted version.

## Funding

This research was partially funded by an unrestricted grant by Lilly S.A. The funder was not involved in the study design, collection, analysis, interpretation of data, the writing of this article or the decision to submit it for publication. This research was supported by CIBER-Consorcio Centro de Investigación Biomédica en Red (leading group: CIBERDEM - CB15/00071), Instituto de Salud Carlos III, Ministerio de Ciencia e Innovación and Unión Europea – European Regional Development Fund.

## Acknowledgments

We thank Amanda Prowse for her support in linguistics.

## Conflict of interest

IG received support for attending meetings form Boehringer Ingelheim, Lilly, MSD, Novo-Nordisk and Sanofi. JF-N received advisory and/or speaking fees from Astra-Zeneca, Ascensia, Boehringer Ingelheim, GSK, Lilly, MSD, Novartis, Novo Nordisk and Sanofi. Received research grants to the institution from Astra-Zeneca. GSK, Lilly, MSD, Novartis, Novo Nordisk, Sanofi and Boehringer. DM has received advisory and/or speaking fees from Almirall, Esteve, Ferrer, Janssen, Lilly, Menarini, MSD, NovoNordisk and Sanofi. GG-P has received speaking fees from Lilly and Astra-Zeneca. MM-C has received advisory honorarium from for Astra-Zeneca, Bayer, Boehringer Ingelheim, GSK, Lilly, MSD, NOVARTIS, NovoNordisk, Sanofi; speaker honorarium from Astra- Zeneca, Bayer, Boehringer Ingelheim, GSK, Lilly, Menarini, MSD, Novartis, NovoNordisk, and Sanofi; and research grants to institution from Astra-Zeneca, GSK, Lilly, MSD, Novartis, NovoNordisk, and Sanofi.

The remaining authors declare that the research was conducted in the absence of any commercial or financial relationships that could be construed as a potential conflict of interest.

## Publisher’s note

All claims expressed in this article are solely those of the authors and do not necessarily represent those of their affiliated organizations, or those of the publisher, the editors and the reviewers. Any product that may be evaluated in this article, or claim that may be made by its manufacturer, is not guaranteed or endorsed by the publisher.
