# Supplementary material for: Obesity and related comorbidities in a large population-based cohort of subjects with type 1 diabetes in Catalonia
Source: Front Endocrinol (Lausanne). 2022 Dec 2;13:1015614. doi: 10.3389/fendo.2022.1015614 (PMC9756841; doi:10.3389/fendo.2022.1015614)
Supplement: Supplementary file 1 [file DataSheet_1.docx]

**SUPPLEMENTARY MATERIAL**

**Appendix 1: ICD-10 diagnostic codes and ATC /DDD codes of the drugs:**

ICD-10 DIAGNOSTIC CODES:

1. Type 1 diabetes mellitus:

E10 - Diabetis mellitus insulinodependent

E10.0 - Diabetis mellitus insulinodependent, amb coma

E10.1 - Diabetis mellitus insulinodependent, amb cetoacidosi

E10.2 - Diabetis mellitus insulinodependent, amb complicacions renals

E10.3 - Diabetis mellitus insulinodependent, amb complicacions oftalmològiques

E10.4 - Diabetis mellitus insulinodependent, amb complicacions neurològiques

E10.5 - Diabetis mellitus insulinodependent, amb complicacions circulatòries perifèriques

E10.6 - Diabetis mellitus insulinodependent, amb altres complicacions específiques

E10.7 - Diabetis mellitus insulinodependent, amb complicacions múltiples

E10.8 - Diabetis mellitus insulinodependent, amb complicacions no específiques

E10.9 - Diabetis mellitus insulinodependent, sense menció de complicacions

1. Hypertension

I10 - Hipertensió essencial (primària)

I11 - Malaltia cardíaca hipertensiva

I11.0 - Malaltia cardíaca hipertensiva amb insuficiència cardíaca (congestiva)

I11.9 - Malaltia cardíaca hipertensiva sense insuficiència cardíaca (congestiva)

I12 - Malaltia renal hipertensiva

I12.0 - Malaltia renal hipertensiva amb insuficiència renal

I12.9 - Malaltia renal hipertensiva sense insuficiència renal

I13 - Malaltia cardiorenal hipertensiva

I13.0 - Malaltia cardiorenal hipertensiva amb insuficiència cardíaca (congestiva)

I13.1 - Malaltia cardiorenal hipertensiva amb insuficiència renal

I13.2 - Malaltia cardiorenal hipertensiva amb insuficiència cardíaca (congestiva) i insuficiència renal

I13.9 - Malaltia cardiorenal hipertensiva, no especificada

I15 - Hipertensió secundària

I15.0 - Hipertensió renovascular

I15.1 - Hipertensió secundària a altres trastorns renals

I15.2 - Hipertensió secundària a trastorns endocrins

I15.8 - Altres tipus d'hipertensió secundària

I15.9 - Hipertensió secundària, no especificada

1. Dyslipidemia:

E78 - Trastorns del metabolisme de les lipoproteïnes i altres lipidèmies

E78.0 - Hipercolesterolèmia pura

E78.1 - Hipergliciridèmia pura

E78.2 - Hiperlipidèmia mixta

E78.3 - Hiperquilomicronèmia

E78.4 - Altres hiperlipidèmies

E78.5 - Hiperlipidèmia no especificada

E78.6 - Deficiència de lipoproteïnes

E78.8 - Altres trastorns del metabolisme de les lipoproteïnes

E78.9 - Trastorn del metabolisme de les lipoproteïnes, no especificat

1. Chronic kindey disease:

E10.2 - Diabetis mellitus insulinodependent, amb complicacions renals

ATC/DDD DRUGS CODES

1. Insulins

*Intermediate-acting insulins:*

A10AC01 - Insulina (humana)

A10AC02 - Insulina (bovina)

A10AC03 - Insulina (porcina)

A10AC04 - Insulina lispro protamina

*Mixed insulins*:

A10AD01 - Insulina (humana)

A10AD02 - Insulina (bovina)

A10AD03 - Insulina (porcina)

A10AD04 - Insulina lispro

A10AD05 - Insulina asparta

A10AD06 - Insulina degludec e insulina asparta

*Long-acting insulins:*

A10AE01 - Insulina (humana)

A10AE02 - Insulina (bovina)

A10AE03 - Insulina (porcina)

A10AE04 - Insulina glargina

A10AE05 - Insulina detemir

A10AE06 - Insulina degludec

*Short-acting insulins:*

A10AB01 - Insulina (humana)

A10AB02 - Insulina (bovina)

A10AB03 - Insulina (porcina)

A10AB04 - Insulina lispro

A10AB05 - Insulina asparta

A10AB06 - Insulina glulisina

*Insulin combinations:*

A10AB30 - Combinaciones

A10AC30 - Combinaciones

A10AD30 - Combinaciones

A10AE30 - Combinaciones

A10AE54 - Insulina glargina y lixisenatida

A10AE56 - Insulina degludec y liraglutida

1. Other anti-diabetic drugs

A10BA01 – Fenformina; A10BA02 – Metformina; A10BA03 – Buformina; A10BB01 – Glibenclamida; A10BB02 – Clorpropamida; A10BB03 – Tolbutamida; A10BB04 – Glibornurida; A10BB05 – Tolazamida; A10BB06 – Carbutamida; A10BB07 – Glipizida; A10BB08 – Gliquidona; A10BB09 – Gliclazida; A10BB10 – Metahexamida; A10BB11 – Glisoxepida; A10BB12 – Glimepirida; A10BB31 – Acetohexamida; A10BB91 – Glisentida; A10BC01 – Glimidina; A10BD01 - Fenformina y sulfonamides; A10BD02 - Metformina y sulfonamides; A10BD03 - Metformina y rosiglitazona; A10BD04 - Glimepirida y rosiglitazona; A10BD05 - Metformina y pioglitazona; A10BD06 - Glimepirida y pioglitazona; A10BD07 - Metformina y sitagliptina; A10BD08 - Metformina y vildagliptina; A10BD09 - Pioglitazona y alogliptina; A10BD10 - Metformina y Saxagliptina; A10BD11 - Metformina y linagliptina; A10BD13 - Metformina y Alogliptina; A10BD15 - Metformina y Dapagliflozina; A10BD16 - Metformina y canagliflozina; A10BD19 - Linagliptina y Empagliflozina; A10BD20 - Metformina y Empagliflozina; A10BD21 - Saxagliptina y Dapagliflozina; A10BF01 – Acarbosa; A10BF02 – Miglitol; A10BF03 – Voglibosa; A10BG01 – Troglitazona; A10BG02 – Rosiglitazona; A10BG03 – Pioglitazona; A10BH01 – Sitagliptina; A10BH02 – Vildagliptina; A10BH03 – Saxagliptina; A10BH04 – Alogliptina; A10BH05 – Linagliptina; A10BJ01 – Exenatida; A10BJ02 – Liraglutida; A10BJ03 – Lixisenatida; A10BJ04 – Albiglutida; A10BJ05 – Dulaglutida; A10BK01 – Dapagliflozina; A10BK02 – Canagliflozina; A10BK03 – Empagliflozina; A10BX01 - Goma guar; A10BX02 – Repaglinida; A10BX03 – Nateglinida; A10BX04 – Exenatida; A10BX05 – Pramlintida; A10BX06 – Benfluorex; A10BX07 – Liraglutida; A10BX08 – Mitiglinida; A10BX09 – Dapagliflozina; A10BX10 – LIXISENATIDA; A10BX11 – Canagliflozina; A10BX12 – Empagliflozina; A10BX13 – Albiglutida; A10BX14 – Dulaglutida

1. Blood pressure lowering agents:

C02AA01 – Rescinamina; C02AA02 – Reserpina; C02AA03 - Alcaloides de la rauwolfia, combinaciones de; C02AA04 - Alcaloides de la rauwolfia, raiz entera de; C02AA05 – Deserpidina;C02AA06 – Metoserpidina; C02AA07 – Bietaserpina; C02AA52 - Reserpina, combinaciones con; C02AA53 - Combinaciones de alcaloides de la rauwolfia, combinaciones con; C02AA57 - Bietaserpina, combinaciones con; C02AB01 - Metildopa (levogira); C02AB02 - Metildopa (racemica); C02AC01 – Clonidina; C02AC02 – Guanfacina; C02AC04 – Tolonidina; C02AC05 – Moxonidina; C02AC06 – Rilmenidina; C02BA01 – Trimetafan; C02BB01 – Mecamilamina; C02CA01 – Prazosina; C02CA02 – Indoramina; C02CA03 – Trimazosina; C02CA04 – Doxazosina; C02CA05 – Terazosina; C02CA06 – Urapidil; C02CC01 – Betanidina; C02CC02 – Guanetidina; C02CC03 – Guanoxano; C02CC04 – Debrisoquina; C02CC05 – Guanocloro; C02CC06 – Guanazodina; C02CC07 – Guanoxabenzo; C02DA01 – Diazoxido; C02DB01 – Dihidralazina; C02DB02 – Hidralazina; C02DB03 – Endralazina; C02DB04 – Cadralazina; C02DB91 - Picodralazina, combinaciones con; C02DC01 – Minoxidil; C02DD01 – Nitroprusiato; C02DG01 – Pinacidil; C02KA01 – Veratro; C02KB01 – Metirosina; C02KC01 – Pargilina; C02KD01 – Ketanserina; C02KX01 – Bosentano; C02KX02 – Ambrisentan; C02KX03 – Sitaxentan; C02KX04 – Macitentan; C02KX05 – Riociguat; C02LA01 - Reserpina y diureticos; C02LA02 - Rescinamina y diureticos; C02LA03 - Deserpidina y diureticos; C02LA04 - Metoserpidina y diureticos; C02LA07 - Bietaserpina y diureticos; C02LA08 - Rauwolfia, alcaloides de raiz entera, y diureticos; C02LA09 - Sirosingopina y diureticos; C02LA50 - Combinacion de alcaloides de la rauwolfia y diureticos, incluyendo otras combinacions; C02LA51 - Reserpina y diureticos, combinaciones con otras farmacos; C02LA52 - Rescinamina y diureticos, combinaciones con otras farmacos; C02LA71 - Reserpina y diureticos, combinaciones con psicolepticos; C02LA91 - Reserpina y tiazidas, combinaciones con vasodilatadores; C02LB01 - Metildopa (levogira) y diureticos; C02LB91 - Metildopa y diureticos; C02LC01 - Clonidina y diureticos; C02LC05 - Moxonidina y diureticos; C02LC51 - Clonidina y diureticos, combinaciones con otras farmacos; C02LE01 - Prazosina y diureticos; C02LF01 - Guanetidina y diureticos; C02LG01 - Dihidralazina y diureticos; C02LG02 - Hidralazina y diureticos; C02LG03 - Picodralazina y diureticos; C02LG51 - Dihidralazina y diureticos, combinaciones con otras farmacos; C02LG73 - Picodralazina y diureticos, combinaciones con psicolepticos; C02LK01 - Veratro y diureticos; C02LL01 - Pargilina y diureticos; C02LX01 - Pinacidil y diureticos; C03AA01 – Bendroflumetiazida; C03AA02 – Hidroflumetiazida; C03AA03 – Hidroclorotiazida; C03AA04 – Clorotiazida; C03AA05 – Politiazida; C03AA06 – Triclormetiazida; C03AA07 – Ciclopentiazida; C03AA08 – Meticlotiazida; C03AA09 – Ciclotiazida; C03AA13 – Mebutizida; C03AB01 - Bendroflumetiazida y potasio; C03AB02 - Hidroflumetiazida y potasio; C03AB03 - Hidroclorotiazida y potasio; C03AB04 - Clorotiazida y potasio; C03AB05 - Politiazida y potasio; C03AB06 - Triclormetiazida y potasio; C03AB07 - Ciclopentiazida y potasio; C03AB08 - Meticlotiazida y potasio; C03AB09 - Ciclotiazida y potasio; C03AH01 - Clorotiazida, combinaciones con; C03AH02 - Hidroflumetiazida, combinaciones con; C03AX01 - Hidroclorotiazida, combinaciones con; C03AX91 - Teclotiazida, combinaciones con; C03BA02 – Quinetazona; C03BA03 – Clopamida; C03BA04 – Clortalidona; C03BA05 – Mefrusida; C03BA07 – Clofenamida; C03BA08 – Metolazona; C03BA09 – Meticrano; C03BA10 – Xipamida; C03BA11 – Indapamida; C03BA12 – Clorexolona; C03BA13 – Fenquizona; C03BA82 - Clorexolona, combinaciones con psicolepticos; C03BB02 - Quinetazona y potasio; C03BB03 - Clopamida y potasio; C03BB04 - Clortalidona y potasio; C03BB05 - Mefrusida y potasio; C03BB07 - Clofenamida y potasio; C03BC01 – Mersalilo; C03BD01 – Teobromina; C03BX03 – Cicletanina; C03CA01 – Furosemida; C03CA02 – Bumetanida; C03CA03 – Piretanida; C03CA04 – Torasemida; C03CB01 - Furosemida y potasio; C03CB02 - Bumetanida y potasio; C03CC01 - Acido etacrinico; C03CC02 - Acido tienilico; C03CD01 – Muzolimina; C03CX01 – Etozolina; C03DA01 – Espironolactona; C03DA02 - Canrenoato de potasio; C03DA03 – Canrenona; C03DA04 – Eplerenona; C03DB01 – Amilorida; C03DB02 – Triamtereno; C03EA01 - Hidroclorotiazida y agentes ahorradores de potasio; C03EA02 - Triclormetiazida y agentes ahorradores de potasio; C03EA03 - Epitizida y agentes ahorradores de potasio; C03EA04 - Altizida y agentes ahorradores de potasio; C03EA05 - Mebutizida y agentes ahorradores de potasio; C03EA06 - Clortalidona y agentes ahorradores de potasio; C03EA07 - Ciclopentiazida y agentes ahorradores de potasio; C03EA12 - Metolazona y agentes ahorradores de potasio; C03EA13 - Bendroflumetiazida y agentes ahorradores de potasio; C03EA14 - Butizida y agentes ahorradores de potasio; C03EB01 - Furosemida y agentes ahorradores de potasio; C03EB02 - Bumetanida y agentes ahorradores de potasio; C03XA01 – Tolvaptan; C03XA02 – Conivaptan; C03XW91 – Urea; C07AA01 – Alprenolol; C07AA02 – Oxprenolol; C07AA03 – Pindolol; C07AA05 – Propranolol; C07AA06 – Timolol; C07AA07 – Sotalol; C07AA12 – Nadolol; C07AA14 – Mepindolol; C07AA15 – Carteolol; C07AA16 – Tertatolol; C07AA17 – Bopindolol; C07AA19 – Bupranolol; C07AA23 – Penbutolol; C07AA27 – Cloranolol; C07AA57 - Sotalol, envases combinados; C07AB01 – Practolol; C07AB02 – Metoprolol; C07AB03 – Atenolol; C07AB04 – Acebutolol; C07AB05 – Betaxolol; C07AB06 – Bevantolol; C07AB07 – Bisoprolol; C07AB08 – Celiprolol; C07AB09 – Esmolol; C07AB10 – Epanolol; C07AB11 – Esatenolol; C07AB12 – Nebivolol; C07AB13 – Talinolol; C07AB52 - Metoprolol, envases combinados; C07AB57 - Bisoprolol, combinaciones con; C07AG01 – Labetalol; C07AG02 – Carvedilol; C07BA02 - Oxprenolol y tiazidas; C07BA05 - Propranolol y tiazidas; C07BA06 - Timolol y tiazidas; C07BA07 - Sotalol y tiazidas; C07BA12 - Nadolol y tiazidas; C07BA68 - Metipranolol y tiazidas, combinaciones con; C07BA91 - Propranolol, bendroflumetiazida e hidralazina, combinacions; C07BB02 - Metoprolol y tiazidas; C07BB03 - Atenolol y tiazidas; C07BB04 - Acebutolol y tiazidas; C07BB06 - Bevantolol y tiazidas; C07BB07 - Bisoprolol y tiazidas; C07BB12 - Nebivolol y tiazidas; C07BB52 - Metoprolol y tiazidas, combinaciones con; C07BB91 - Atenolol, bendroflumetiazida e hidralazina, combinaciones co; C07BG01 - Labetalol y tiazidas; C07CA02 - Oxprenolol y otros diureticos; C07CA03 - Pindolol y otros diureticos; C07CA17 - Bopindolol y otros diureticos; C07CA23 - Penbutolol y otros diureticos; C07CB02 - Metoprolol y otros diureticos; C07CB03 - Atenolol y otros diureticos; C07CB53 - Atenolol y otros diureticos, combinaciones con; C07CG01 - Labetalol y otros diureticos; C07DA06 - Timolol, tiazidas y otros diureticos; C07DB01 - Atenolol, tiazidas y otros diureticos; C07EA91 - Propranolol asociado a hidralazina; C07FA05 - Propranolol y otros antihipertensivos; C07FB02 - Metoprolol y felodipino; C07FB03 - Atenolol y nifedipino; C07FB07 - Bisoprolol y amlodipino; C07FX01 - Propranolol y otras combinacions; C07FX06 - Carvedilol e Ivabradina; C08CA01 – Amlodipino; C08CA02 – Felodipino; C08CA03 – Isradipino; C08CA04 – Nicardipino; C08CA05 – Nifedipino; C08CA06 – Nimodipino; C08CA07 – Nisoldipino; C08CA08 – Nitrendipino; C08CA09 – Lacidipino; C08CA10 – Nilvadipino; C08CA11 – Manidipino; C08CA12 – Barnidipino; C08CA13 – Lercanidipino; C08CA14 – Cilnidipino; C08CA15 – Benidipino; C08CA16 – Clevidipino; C08CA55 - Nifedipino, combinaciones con; C08CX01 – Mibefradil; C08DA01 – Verapamilo; C08DA02 – Galopamilo; C08DA51 - Verapamilo, combinaciones con; C08DB01 – Diltiazem; C08EA01 – Fendilina; C08EA02 – Bepridil; C08EX01 – Lidoflazina; C08EX02 – Perhexilina; C08GA01 - Nifedipino y diureticos; C08GA02 - Amlodipino y diureticos; C09AA01 – Captopril; C09AA02 – Enalapril; C09AA03 – Lisinopril; C09AA04 – Perindopril; C09AA05 – Ramipril; C09AA06 – Quinapril; C09AA07 – Benazepril; C09AA08 – Cilazapril; C09AA09 – Fosinopril; C09AA10 – Trandolapril; C09AA11 – Espirapril; C09AA12 – Delapril; C09AA13 – Moexipril; C09AA14 – Temocapril; C09AA15 – Zofenopril; C09AA16 – Imidapril; C09BA01 - Captopril y diureticos; C09BA02 - Enalapril y diureticos; C09BA03 - Lisinopril y diureticos; C09BA04 - Perindopril y diureticos; C09BA05 - Ramipril y diureticos; C09BA06 - Quinapril y diureticos; C09BA07 - Benazepril y diureticos; C09BA08 - Cilazapril y diureticos; C09BA09 - Fosinopril y diureticos; C09BA12 - Delapril y diureticos; C09BA13 - Moexipril y diureticos; C09BA15 - Zofenopril y diureticos; C09BB02 - Enalapril y lercanidipino; C09BB03 - Lisinopril y amlodipino; C09BB04 - Perindopril y amlodipino; C09BB05 - Ramipril y felodipino; C09BB06 - Enalapril y nitrendipino; C09BB07 - Ramipril y amlodipino; C09BB10 - Trandolapril y verapamilo; C09BB12 - Delapril y manidipino; C09BX01 - Perindopril/Amlodipino/Indapamida; C09CA01 – Losartan; C09CA02 – Eprosartan; C09CA03 – Valsartan; C09CA04 – Irbesartan; C09CA05 – Tasosartan; C09CA06 – Candesartan; C09CA07 – Telmisartan; C09CA08 - Olmesartan medoxomilo; C09CA09 - Azilsartan medoxomilo; C09DA01 - Losartan y diureticos; C09DA02 - Eprosartan y diureticos; C09DA03 - Valsartan y diureticos; C09DA04 - Irbesartan y diureticos; C09DA06 - Candesartan y diureticos; C09DA07 - Telmisartan y diureticos; C09DA08 - Olmesartan medoxomilo y diureticos; C09DB01 - Valsartan y Amlodipino; C09DB02 - Olmesartan medoxomilo y amlodipino; C09DB04 - Telmisartan y amlodipino; C09DB05 - Irbesartan y amlodipino; C09DB06 - Losartan y amlodipino; C09DB07 - Candesartan y amlodipino; C09DX01 - Valsartan, amlodipino e hidroclorotiazida; C09DX02 - Valsartan y aliskireno; C09DX03 - Olmesartan medoxomilo, amlodipino e hidroclorotiazida; C09DX04 - Valsartan y sacubitrilo; C09XA01 – Remikireno; C09XA02 – Aliskireno; C09XA52 - Aliskireno e hidroclorotiazida; C09XA53 - Aliskireno y amlodipino; C09XA54 - Aliskireno, amlodipino e hidroclorotiazida

1. Antitrombic agents:

B01AA01 – Dicumarol; B01AA02 – Fenindiona; B01AA03 – Warfarina; B01AA04 – Fenprocumon; B01AA07 – Acenocumarol, B01AA08 - Biscumacetato de etilo; B01AA09 – Clorindiona; B01AA10 – Difenadiona; B01AA11 – Tioclomarol; B01AA12 – Fluindiona; B01AB01 – Heparina; B01AB02 - Antitrombina III; B01AB04 – Dalterapina; B01AB05 – Enoxaparina; B01AB06 – Nadroparina; B01AB07 – Parnaparina; B01AB08 – Reviparina; B01AB09 – Danaparoid; B01AB10 – Tinzaparina; B01AB11 – Sulodexida; B01AB12 – Bemiparina; B01AB51 - Heparina, combinaciones de; B01AC01 – Ditazol; B01AC02 – Cloricromeno; B01AC03 – Picotamida; B01AC04 – Clopidogrel; B01AC05 – Ticlopidina; B01AC06 - Acido acetilsalicilico; B01AC07 – Dipiridamol; B01AC08 - Carbasalato de calcio; B01AC09 – Epoprostenol; B01AC10 – Indobufen; B01AC11 – Iloprost; B01AC13 – Abciximab; B01AC14 – Anagrelida; B01AC15 – Aloxiprina; B01AC16 – Eptifibatida; B01AC17 – Tirofiban; B01AC18 – Triflusal; B01AC19 – Beraprost; B01AC21 – Treprostinilo; B01AC22 – Prasugrel; B01AC23 – Cilostazol; B01AC24 – Ticagrelor; B01AC26 – Vorapaxar; B01AC27 – Selexipag; B01AC30 – Combinaciones; B01AC56 - Acido acetilsalicilico y esomeprazol; B01AC91 - Acetilsaliciclico acido y dipiridamol, combinaciones con; B01AC93 – Sulfinpirazona; B01AD01 – Estreptoquinasa; B01AD02 – Alteplasa; B01AD03 – Anistreplasa; B01AD04 – Uroquinasa; B01AD05 – Fibrinolisina; B01AD06 – Brinasa; B01AD07 – Reteplasa; B01AD08 – Saruplasa; B01AD09 – Ancrod; B01AD10 - Drotrecogina alfa (activado); B01AD11 – Tenecteplasa; B01AD12 - Proteina C; B01AE01 – Desirudina; B01AE02 – Lepirudina; B01AE03 – Argatroban; B01AE04 – Melagatran; B01AE05 – Ximelagatran; B01AE06 – Bivalirudina; B01AE07 - Dabigatran etexilato; B01AF01 – Rivaroxaban; B01AF02 – Apixaban; B01AF03 – Edoxaban; B01AX01 – Defibrotida; B01AX02 – Disuridina; B01AX03 – Lepirudina; B01AX04 - Dermatan, sulfato de; B01AX05 – Fondaparinux; B01AX06 – Rivaroxaban; B01AX91 - Proteina C humana

1. Lipid-lowering agents

A12CC10 - Oxido de magnesio; C10AA01 – Simvastatina; C10AA02 – Lovastatina; C10AA03 – Pravastatina; C10AA04 – Fluvastatina; C10AA05 – Atorvastatina; C10AA06 – Cerivastatina; C10AA07 – Rosuvastatina; C10AA08 – Pitavastatina; C10AA51 - Simvastatina, envases combinados; C10AA52 - Lovastatina, combinacions; C10AA53 - Pravastatina, combinacions; C10AA55 - Atorvastatina, combinacions; C10AB01 – Clofibrato; C10AB02 – Bezafibrato; C10AB03 - Clofibrato aluminio; C10AB04 – Genfibrozilo; C10AB05 – Fenofibrato; C10AB06 – Sinfibrato; C10AB07 – Ronifibrato; C10AB08 – Ciprofibrato; C10AB09 – Etofibrato; C10AB10 – Clofibrida; C10AB11 - Fenofibrato de colina; C10AB51 - Clofibrato, combinaciones con; C10AB53 - Clofibrato de aluminio, combinaciones con; C10AB91 – Binifibrato; C10AB92 – Plafibrida; C10AB93 – Pirifibrato; C10AB94 - Clofibrato y piricarbato, combinaciones con; C10AB96 – Tocofibrato; C10AB97 - Clofibrato de aluminio y piricarbato, combinaciones con; C10AB98 – Nicofibrato; C10AC01 – Colestiramina; C10AC02 – Colestipol; C10AC03 – Colextran; C10AC04 – Colesevelam; C10AC91 – Filicol; C10AD01 – Niceritrol; C10AD02 - Acido nicotinico; C10AD03 – Nicofuranosa; C10AD04 - Nicotinato de aluminio; C10AD05 - Nicotinil alcohol (piridilcarbinol); C10AD06 – Acipimox; C10AD52 - Acido nicotinico, combinaciones con; C10AD91 - Nicotinato de tocoferol; C10AD92 - Nicotinato de tocoferol, combinaciones con; C10AD93 – Piricarbato; C10AD94 - Piricarbato, combinaciones con; C10AD95 – Pirozadilo; C10AD96 – Nicofibrato; C10AD97 – Nicoclonato; C10AD98 - Nicotinico acido y pentosanopolisulfurico, combinaciones con; C10AD99 - Nicotinato xantinol y mucopolisacarido, combinaciones con; C10AX01 – Dextrotiroxina; C10AX02 – Probucol; C10AX03 – Tiadenol; C10AX04 – Benfluorex; C10AX05 – Meglutol; C10AX06 - Trigliceridos omega-3, incluidos otros esteres y acidos; C10AX07 - Magnesio piridoxal 5-fosfato glutamato; C10AX08 – Policosanol; C10AX09 – Ezetimiba; C10AX10 - Alipogen tiparvovec; C10AX11 – Mipomersen; C10AX12 – Lomitapida; C10AX13 – Evolocumab; C10AX14 – Alirocumab; C10AX90 – Dextrotirosina; C10AX91 – Sulodexida; C10AX93 – Pantetina; C10AX94 – Heparinoide; C10AX95 - Sultosilato de piperazina; C10AX97 - Amilasa, lipasa y tirosinasa, combinaciones con; C10AX99 - Fosfolipidos, combinaciones con; C10BA01 - Lovastatina y acido nicotinico; C10BA02 - Simvastatina y ezetimiba; C10BA03 - Pravastatina y fenofibrato; C10BA04 - simvastatina y fenofibrato; C10BA05 - Atorvastatina y Ezetimiba; C10BA06 - Rosuvastatina y Ezetimiba; C10BX01 - Simvastatina y acido acetilsalicilico; C10BX02 - Pravastatina y acido acetilsalicilico; C10BX03 - Atorvastatina y Amlodipino; C10BX04 - Simvastatina, acido acetilsalicilico y ramipril; C10BX06 - Atorvastatina, acido acetilsalicilico y ramipril; C10BX09 - Rosuvastatina y amlodipino
